# Finding flow at the piano: psychological regulation and configurational profiles among art students at Chinese sport universities

**DOI:** 10.3389/fpsyg.2026.1886877

**Published:** 2026-08-03

**Authors:** Yaqing Zhang

**Affiliations:** School of Arts, Chengdu Sport University, Chengdu, China

**Keywords:** art students, CB-SEM, cognitive emotion regulation, fsQCA, mindfulness, piano performance flow, self-control, sport universities

## Abstract

**Purpose:**

This study examined whether general self-reported psychological regulatory resources were associated with retrospectively reported piano performance flow among art students at Chinese sport universities, focusing on mindfulness, self-control, and adaptive and maladaptive cognitive emotion regulation.

**Methods:**

A cross-sectional survey was conducted with 1,476 art students from 10 Chinese sport universities. Piano performance flow was measured using a piano-performance-adapted Chinese Work-related Flow Inventory, with current-sample CFA-based measurement evidence reported before structural testing. Mindfulness, self-control, and cognitive emotion regulation were assessed using validated Chinese instruments. Structural equation modeling examined direct and indirect associations, while fuzzy-set qualitative comparative analysis identified configurations associated with high and non-high flow.

**Results:**

Mindfulness was positively associated with self-control and piano performance flow. Self-control was positively associated with adaptive cognitive emotion regulation and negatively associated with maladaptive cognitive emotion regulation. Adaptive cognitive emotion regulation was positively associated with flow, whereas maladaptive cognitive emotion regulation was negatively associated with it. The direct association between self-control and flow was not significant after adjustment. Significant indirect associations were found through self-control and both forms of cognitive emotion regulation. fsQCA indicated that no single condition was necessary for high or non-high flow. High flow was associated with two configurations combining high mindfulness and low maladaptive cognitive emotion regulation with either high self-control or high adaptive cognitive emotion regulation. Non-high flow was associated with low mindfulness, low adaptive cognitive emotion regulation, and high maladaptive cognitive emotion regulation.

**Conclusion:**

Piano performance flow in this sport-university context was associated with psychological regulatory resources and configurations of those resources. These cross-sectional findings should be interpreted as correlational and hypothesis-generating. They identify candidate psychological resources for future performance-education research rather than demonstrating causal, real-time, or intervention effects.

## 1 Introduction

### 1.1 Piano performance flow as a fragile optimal experience

Piano performance is not only a technical act of producing accurate notes (Li et al., 2024). It is a demanding performance situation in which students must coordinate motor execution, auditory monitoring, memory retrieval, expressive intention, emotional arousal, attentional focus, and self-evaluation under real or imagined audience pressure (De Manzano et al., 2010; Jha et al., 2022). In such a situation, a performer may experience an optimal state in which attention becomes highly concentrated, action feels smooth, self-conscious monitoring decreases, and the performance itself becomes intrinsically rewarding (Marin and Bhattacharya, 2013). This state is commonly understood through the concept of flow (Csikszentmihalyi, 1990).

Flow theory describes flow as an optimal experience characterized by deep involvement, intense concentration, intrinsic enjoyment, a sense of control, and reduced self-consciousness when a person is fully engaged in an activity (Csikszentmihalyi, 1990; Nakamura and Csikszentmihalyi, 2002). In music performance, flow is especially meaningful because successful performance requires more than technical accuracy. The performer must remain immersed in the musical task while managing bodily tension, emotional expression, performance pressure, and possible mistakes. Empirical studies on musicians have shown that flow is closely related to emotional functioning, self-efficacy, coping, musical training, anxiety, and performance-related psychological characteristics (Marin and Bhattacharya, 2013; Rakei et al., 2022; Spahn et al., 2021). These findings suggest that piano performance flow is not simply a spontaneous pleasant state; rather, it is an optimal but fragile performance experience that may be closely related to how students manage attention, action, and emotion in performance-related contexts.

In the present study, piano performance flow experience is conceptualized as a retrospectively reported short-term optimal experience involving absorption, enjoyment, and intrinsic motivation (Bakker, 2008). This conceptualization is informed by Bakker’s (2008) Work-related Flow Inventory (WOLF), which defines flow as a peak experience consisting of absorption, work enjoyment, and intrinsic work motivation. Although WOLF was originally developed in occupational settings, these three components capture experiential qualities that are also central to performance-based flow: being absorbed in the act of playing, enjoying the performance process, and being motivated by the musical activity itself. The Chinese version of WOLF has also supported a three-factor structure consisting of absorption, enjoyment, and intrinsic motivation (Chen et al., 2016; Kang, 2022). Accordingly, the present study uses a piano-performance-adapted version of the WOLF framework as an operational measure of recalled piano performance flow experience and examines its measurement properties in the current sample before analyzing its associations with psychological regulatory resources.

### 1.2 Why art students at Chinese sport universities matter

Art students at Chinese sport universities represent a meaningful but underexamined group for studying piano performance flow. They are not simply music students placed in a different institutional setting. Their artistic learning is embedded in an educational ecology traditionally associated with physical education, sport science, bodily discipline, competition, performance assessment, and embodied training (Pineda-Espejel et al., 2020; Uzun Dönmez and Imamoglu, 2020). Compared with students in specialized conservatories, art students at sport universities may experience piano learning and performance within a broader institutional environment that emphasizes body control, discipline, evaluation, and performance under pressure (Draugelis et al., 2014; Pineda-Espejel et al., 2020).

This context makes their piano performance experience theoretically interesting. Piano performance requires fine motor coordination, auditory sensitivity, emotional expression, and aesthetic interpretation (Şuteu, 2024). Sport-university training contexts, by contrast, often emphasize bodily regulation, persistence, self-management, and performance readiness (Hatfield, 2016). The intersection of these two domains provides a meaningful setting for examining psychological regulation in piano performance. In the present study, Chinese sport universities are treated as a bounded institutional context rather than as a directly measured explanatory variable. This framing allows the study to focus on how psychological regulatory resources are associated with piano performance flow among art students in this particular educational setting.

Existing research on flow in music has mainly focused on professional musicians, conservatory students, orchestral performers, or general music learners. Studies have shown that music performance flow is negatively related to anxiety and positively related to functional coping, self-efficacy, emotional intelligence, and musical training (Marin and Bhattacharya, 2013; Rakei et al., 2022; Spahn et al., 2021). However, less is known about piano performance flow among art students in Chinese sport universities, especially from the perspective of general psychological regulatory resources. This study addresses this gap by examining whether mindfulness, self-control, and cognitive emotion regulation are associated with students’ recalled piano performance flow experience in this sport-university context.

### 1.3 Psychological regulation as a basis for piano performance flow

Flow is often described as effortless, but the conditions associated with it are not necessarily effortless (Srinivasan and Gingras, 2014). In piano performance, students need to regulate attention, inhibit distraction, manage performance-related thoughts, and respond to mistakes or evaluative pressure. Psychological regulation therefore provides a useful theoretical lens for understanding individual differences in piano performance flow (Baltazar and Saarikallio, 2019).

The first regulatory resource considered in this study is mindfulness. Mindfulness refers to present-moment awareness and the capacity to attend to experience without immediately judging or reacting to it. The Five Facet Mindfulness Questionnaire conceptualizes mindfulness as a multidimensional construct including observing, describing, acting with awareness, non-judging of inner experience, and non-reactivity to inner experience (Baer et al., 2006). The Chinese version of the FFMQ has also shown acceptable psychometric properties in Chinese student samples (Deng et al., 2011). In piano performance contexts, mindfulness may be relevant because it reflects a general tendency to notice bodily sensations, sound quality, emotional tension, and performance-related thoughts while remaining engaged with the ongoing task. A meta-analysis has further shown a positive association between mindfulness and flow, suggesting that individuals with higher mindfulness tend to report stronger flow experiences (Schutte and Malouff, 2023).

The second regulatory resource is self-control. Self-control refers to the capacity to regulate impulses, resist distractions, maintain goal-directed behavior, and act in accordance with longer-term aims (Tangney et al., 2004). The Chinese revision of the Self-Control Scale has identified dimensions such as impulse control, healthy habits, resisting temptation, focused work, and moderation in entertainment among Chinese college students (Tan and Guo, 2008). In piano performance, self-control should not be understood as excessive conscious control over every movement during playing, because over-monitoring may interrupt fluent performance. Rather, self-control can be understood as a broader preparatory and regulatory resource. It is relevant to practice discipline, distraction management, impulse regulation, and the maintenance of task focus in performance-related learning contexts.

The third regulatory component is cognitive emotion regulation. Emotion regulation refers to the processes through which individuals influence the emotions they have, when they have them, and how they experience or express them (Gross, 1998). Cognitive emotion regulation focuses specifically on the cognitive strategies individuals use after stressful or negative events (Garnefski et al., 2001). In performance contexts, students may respond to mistakes, audience pressure, or evaluation concerns in different ways. Adaptive cognitive emotion regulation strategies, such as acceptance, positive refocusing, refocus on planning, positive reappraisal, and putting into perspective, may help students reinterpret performance difficulties and redirect attention to the musical task. Maladaptive strategies, such as self-blame, rumination, catastrophizing, and other-blame, may heighten self-consciousness, disrupt concentration, and weaken flow. The Chinese version of the Cognitive Emotion Regulation Questionnaire has supported the assessment of these cognitive strategies among Chinese university students (Zhu et al., 2007).

Taken together, mindfulness, self-control, and cognitive emotion regulation form a coherent psychological regulatory framework. Mindfulness reflects present-moment awareness and non-reactivity; self-control reflects goal-directed attention and behavioral regulation; cognitive emotion regulation reflects how students cognitively interpret stressful or evaluative experiences. In the present study, these constructs are treated as general self-reported regulatory tendencies rather than momentary regulatory states captured during performance. This framework suggests that piano performance flow is unlikely to be characterized by one isolated psychological trait alone. Instead, recalled piano performance flow may be associated with patterns of attentional awareness, self-regulation, and cognitive emotion regulation.

### 1.4 A theory-informed net-association model of dual emotion-regulation correlates

The first analytical purpose of this study is to examine the net-association relationships among mindfulness, self-control, cognitive emotion regulation, and piano performance flow experience. The proposed model assumes that mindfulness is positively associated with self-control, and that self-control is associated with two contrasting forms of cognitive emotion regulation: adaptive cognitive emotion regulation and maladaptive cognitive emotion regulation. These two forms of cognitive emotion regulation are expected to show opposite associations with piano performance flow experience.

This model is not intended to reduce psychological regulation to a simple one-directional chain. Rather, it organizes the study variables into a theory-informed association structure. On the one hand, mindfulness may be associated with stronger self-control, which in turn may be linked to more adaptive cognitive emotion regulation and stronger piano performance flow. On the other hand, mindfulness may also be associated with stronger self-control, which may be linked to lower maladaptive cognitive emotion regulation and, in turn, stronger flow experience. In addition, mindfulness may have a direct association with piano performance flow because present-moment awareness and non-reactivity are closely aligned with the attentional qualities of flow. Self-control may also be associated with flow, although its role may be expressed more clearly through emotion-regulation-related processes.

This net-association model allows the study to examine whether the observed covariance pattern is consistent with the proposed psychological regulatory framework. It addresses the question of whether mindfulness, self-control, adaptive cognitive emotion regulation, and maladaptive cognitive emotion regulation are statistically associated with piano performance flow experience through direct and indirect associations. However, average net associations alone may not fully capture how different psychological resources combine across individuals. Students may report high flow in relation to different combinations of regulatory resources, and the profiles associated with high flow may not simply be the opposite of those associated with non-high flow. Therefore, a configurational perspective is also useful.

### 1.5 A configurational view of multiple profiles associated with flow

Performance flow may be associated with configurational complexity. Different students may report high piano performance flow in relation to different combinations of psychological resources. One student may report high flow in the presence of high mindfulness, strong self-control, and low maladaptive cognitive emotion regulation. Another may report high flow in relation to high mindfulness, adaptive cognitive emotion regulation, and low maladaptive cognitive emotion regulation, even if self-control is not the defining condition. Conversely, non-high flow may be associated with low mindfulness and weak adaptive regulation combined with high maladaptive cognitive emotion regulation. These possibilities reflect equifinality and configurational asymmetry: similar experiential states may be linked to more than one profile of conditions, and the profiles associated with the presence of high flow may differ from those associated with its absence.

Fuzzy-set qualitative comparative analysis (fsQCA) is appropriate for examining such configurational patterns. Unlike conventional variance-based approaches, fsQCA treats cases as configurations of conditions and identifies combinations that are sufficient for an outcome (Ragin, 2008). Fiss (2011) further emphasized that configurational methods can help distinguish how core and peripheral conditions combine within theoretically meaningful profiles. More recent methodological work also highlights that fsQCA can complement structural equation modeling by revealing asymmetric and configuration-based explanations that may be overlooked in net-effect analysis (Pappas and Woodside, 2021).

In the present study, fsQCA is used to identify configurations associated with high and non-high piano performance flow experience. The condition sets include mindfulness, self-control, adaptive cognitive emotion regulation, and maladaptive cognitive emotion regulation. Following fsQCA terminology, the study refers to conditions and sufficiency; in the present cross-sectional application, however, the resulting solutions are interpreted as set-theoretic membership relations and configurational profiles associated with piano performance flow. This design allows the study to move beyond the question of whether a single variable has a significant average association and toward the question of which combinations of psychological regulatory conditions characterize high and non-high flow.

### 1.6 Research objectives and contributions

This study examines piano performance flow experience among art students at Chinese sport universities from a psychological regulatory perspective. Specifically, it pursues three objectives. First, it examines the cross-sectional net associations among mindfulness, self-control, adaptive cognitive emotion regulation, maladaptive cognitive emotion regulation, and piano performance flow experience using structural equation modeling. Second, it estimates theory-informed indirect associations through which mindfulness is statistically associated with piano performance flow via self-control and cognitive emotion regulation. Third, it identifies configurational profiles of psychological regulatory conditions associated with high and non-high piano performance flow experience using fsQCA.

The study makes three contributions. Theoretically, it extends discussion of music performance flow by linking recalled piano performance flow with general psychological regulatory resources. Rather than treating flow only as a product of musical skill, personality, or practice experience, the study highlights how attentional awareness, self-control, and cognitive emotion regulation are associated with optimal performance experience. Methodologically, it integrates SEM-based net-association analysis with fsQCA-based configurational analysis, thereby combining average association patterns with set-theoretic profiles of high and non-high flow. Practically, it generates candidate directions for future performance-education and intervention research in sport-university contexts by suggesting that mindful attention, self-regulatory habits, adaptive appraisal, and reduced maladaptive cognitive responses may be useful targets for further empirical testing.

## 2 Method

### 2.1 Participants and procedure

This study adopted a cross-sectional online survey design. Participants were recruited from 10 sport universities in China: Beijing Sport University, Shanghai University of Sport, Chengdu Sport University, Wuhan Sports University, Xi’an Physical Education University, Capital University of Physical Education and Sports, Tianjin University of Sport, Shandong Sport University, Shenyang Sport University, and Guangzhou Sport University. The target participants were art students who had experience with piano learning, piano courses, piano examinations, class performances, recitals, or other piano-related performance activities.

The inclusion criteria were as follows: participants had to be adults, enrolled as art students at one of the participating sport universities, able to understand and complete the Chinese questionnaire independently, and willing to provide written informed consent. Participants who did not meet these criteria were not included in the final analysis. The study was approved by the Ethics Committee of Chengdu Sport University. Data were collected from September 15, 2025, to October 20, 2025. The questionnaire was administered online using the Wenjuanxing platform. Before entering the formal questionnaire, all participants were presented with an informed consent page explaining the study purpose, voluntary nature of participation, approximate completion time, anonymity of responses, confidentiality of data, and the right to withdraw before submission. All participants were adults, and written informed consent was obtained from every participant. Only those who selected the consent option were allowed to proceed to the questionnaire; those who did not provide consent could not continue.

To increase sample diversity, the questionnaire was distributed as far as possible according to a stratified recruitment principle, with consideration given to university, grade, and art-related academic background. Because a complete sampling frame was not available, recruitment was conducted through a combination of snowball-assisted sampling, peer recommendation, and link forwarding to class WeChat groups or other student group chats. Teachers, class representatives, and student contacts from the participating universities helped disseminate the survey link to eligible students. Participants were also encouraged to forward the link to other eligible art students within the 10 target sport universities. The survey was not intended as an unrestricted open online survey; dissemination was limited to eligible art students within the 10 target sport universities. Therefore, the final sample should be understood as a multi-site purposive online sample with snowball-assisted recruitment rather than as a population-representative probability sample.

A total of 1,669 questionnaires were returned. The Wenjuanxing system was set so that all substantive items were mandatory; therefore, there were no missing item-level responses. Before data analysis, responses were screened using pre-specified data-quality criteria. Questionnaires were removed if they met one or more of the following conditions: failure to meet the inclusion criteria, inconsistent demographic information, completion time shorter than 267 s, failure on the attention-check item, clear straight-lining or invariant responding across long sections of the questionnaire, mechanically repeated response patterns, or duplicate/highly similar submissions identified through non-identifying platform-level records such as submission time and device/browser information. An example attention-check item was: “This item is used to check whether you are reading carefully; please select ‘Agree’ for this item.” Responses that did not follow this instruction were excluded. After screening, 193 questionnaires were removed, leaving 1,476 retained questionnaires. This yielded a post-screening retention rate of 88.44% among returned questionnaires. Because recruitment involved link forwarding and no complete invitation denominator was available, this value should not be interpreted as a population response rate.

The final sample size was sufficient for the planned analyses. For the covariance-based structural equation modeling component, the measurement model used dimension-level indicators for five reflective latent constructs: mindfulness, self-control, adaptive cognitive emotion regulation, maladaptive cognitive emotion regulation, and piano performance flow experience. This resulted in 22 manifest indicators. A retained sample of 1,476 substantially exceeds common recommendations for structural equation modeling and provides adequate power and estimation stability for estimating direct and indirect associations. Methodological literature emphasizes that SEM sample size requirements depend on model complexity, factor loadings, distributional characteristics, and estimation method rather than a single universal rule; nevertheless, larger samples improve parameter stability and reduce the likelihood of improper solutions (Wolf et al., 2013; Kline, 2023). The sample was also adequate for the fsQCA component. With four main conditions—mindfulness, self-control, adaptive cognitive emotion regulation, and maladaptive cognitive emotion regulation—the truth table contains 16 logically possible configurations, and the sample size of 1,476 provides sufficient empirical coverage for identifying configurational profiles associated with high and non-high piano performance flow experience.

The demographic and piano-related characteristics of the retained sample, including gender, grade, university, art-related academic track, piano learning experience, weekly piano practice time, and recent piano performance context, are summarized in Table 1. As shown in Table 1, the distribution across the 10 universities ranged from 8.06 to 13.96%, indicating that no single institution dominated the retained sample.

**Table 1:** Demographic characteristics of the sample (*n* = 1,476).

Variable	Category	*n*	%
Gender	Male	560	37.94
	Female	916	62.06
Grade	Year 1	337	22.83
	Year 2	394	26.69
	Year 3	376	25.47
	Year 4	331	22.43
	Postgraduate	38	2.57
University	Beijing Sport University	128	8.67
	Shanghai University of Sport	133	9.01
	Chengdu Sport University	204	13.82
	Wuhan Sports University	157	10.64
	Xi’an Physical Education University	142	9.62
	Capital University of Physical Education and Sports	126	8.54
	Tianjin University of Sport	119	8.06
	Shandong Sport University	136	9.21
	Shenyang Sport University	125	8.47
	Guangzhou Sport University	206	13.96
Art-related academic track	Musicology / Music Performance	438	29.67
	Dance Performance / Choreography	342	23.17
	Sports Dance / Artistic Performance	317	21.48
	Broadcasting, Hosting, and Arts-related Communication	166	11.25
	Other arts-related programs	213	14.43
Piano learning experience	Less than 1 year	189	12.80
	1–3 years	461	31.23
	4–6 years	432	29.27
	More than 6 years	394	26.69
Weekly piano practice time	Less than 2 h	276	18.70
	2–4 h	508	34.42
	5–7 h	416	28.18
	8 h or more	276	18.70
Recent piano performance context	Course examination or classroom performance	681	46.14
	School recital or public performance	519	35.16
	Competition, grading examination, or invited performance	276	18.70

### 2.2 Measurements

#### 2.2.1 Piano performance flow experience

Piano performance flow experience (PFLOW) was measured using a piano-performance-adapted version of the Chinese WOrk-reLated Flow Inventory (WOLF). The original WOLF was developed by Bakker (2008) to assess flow as a short-term optimal experience characterized by absorption, work enjoyment, and intrinsic work motivation. The Chinese version of the WOLF was examined by Chen et al. (2016), who reported a three-factor structure consisting of absorption, enjoyment, and intrinsic motivation. The Chinese WOLF contains 13 items, including absorption (ABS; 4 items), enjoyment (ENJ; 4 items), and intrinsic motivation (IM; 5 items).

Because the present study focuses on piano performance rather than work, item wording was contextually adapted by replacing references to “work” with “piano performance” or “during piano performance.” Participants were asked to report their experience during a recent piano performance, recital, examination performance, or public performance. Items were rated on a five-point Likert scale ranging from 1 = strongly disagree to 5 = strongly agree. Dimension scores were calculated by averaging the items within each dimension, with higher scores indicating stronger piano performance flow experience. Representative adapted items included: “During piano performance, I become fully absorbed in what I am doing” for ABS; “I enjoy the process of piano performance” for ENJ; and “Piano performance itself motivates me” for IM.

Because the WOLF was contextually adapted from a work-related to a piano-performance setting, its measurement properties were examined in the current sample before structural testing. Specifically, a second-order confirmatory factor analysis was specified in which the 13 adapted items loaded onto three first-order factors—ABS, ENJ, and IM—and these three first-order factors then loaded onto the higher-order PFLOW construct. In the CB-SEM model, ABS, ENJ, and IM were used as manifest indicators of the latent variable PFLOW. Thus, the adapted measure was not treated as an unexamined scale. However, the present study does not claim to provide a full validation of a new music-specific flow instrument; rather, it uses a theoretically grounded contextual adaptation and reports current-sample measurement evidence for the adapted PFLOW construct.

#### 2.2.2 Mindfulness

Mindfulness (MIND) was assessed using the Chinese version of the Five Facet Mindfulness Questionnaire (Ch-FFMQ). The original FFMQ was developed by Baer et al. (2006) to measure mindfulness as a multidimensional construct. The Chinese version was validated by Deng et al. (2011) in a non-clinical Chinese student sample, and their confirmatory factor analysis supported the five-factor model.

The Ch-FFMQ contains 39 items and five dimensions: observing (OBS; 8 items), describing (DES; 8 items), acting with awareness (AWA; 8 items), non-judging of inner experience (NJ; 8 items), and non-reactivity to inner experience (NR; 7 items). Items are rated on a five-point Likert scale ranging from 1 = never or very rarely true to 5 = very often or always true. Negatively worded items are reverse-coded before calculating scores. Dimension scores were calculated by averaging the corresponding items, with higher scores indicating a higher level of mindfulness. In the CB-SEM model, OBS, DES, AWA, NJ, and NR were used as manifest indicators of the latent variable MIND.

Representative item content included noticing bodily sensations while walking for OBS, putting feelings into words for DES, doing tasks automatically without awareness for AWA, criticizing oneself for inappropriate emotions for NJ, and noticing distressing thoughts without immediately reacting for NR. The AWA and NJ representative items reflect reverse-coded content.

#### 2.2.3 Self-control

Self-control (SC) was measured using the Chinese revision of the Self-Control Scale (SCS). The original SCS was developed by Tangney et al. (2004) to assess individual differences in self-control. Tan and Guo (2008) revised the SCS for Chinese college students and reported that the revised scale had a five-factor structure with acceptable reliability and validity in a sample of 799 Chinese university students.

The Chinese SCS contains 19 items and five dimensions: impulse control (IC; 6 items), healthy habits (HH; 3 items), resisting temptation (RT; 4 items), focused work (FW; 3 items), and moderation in entertainment (ME; 3 items). Items are rated on a five-point Likert scale ranging from 1 = completely inconsistent to 5 = completely consistent. Reverse-coded items were recoded before score calculation. Dimension scores were calculated by averaging the items within each dimension, with higher scores indicating stronger self-control. In the CB-SEM model, IC, HH, RT, FW, and ME were used as manifest indicators of the latent variable SC.

Representative item content included acting impulsively for IC, difficulty maintaining healthy routines for HH, resisting temptations for RT, sticking to action plans or long-term tasks for FW, and over-engaging in enjoyable but harmful activities for ME. The IC, HH, and ME representative examples mainly reflect reverse-coded content.

#### 2.2.4 Cognitive emotion regulation

Cognitive emotion regulation (CER) was measured using the Chinese version of the Cognitive Emotion Regulation Questionnaire (CERQ-C). The original CERQ was developed by Garnefski et al. (2001) to assess cognitive coping strategies used after negative or stressful events. The CERQ contains 36 items measuring nine cognitive emotion regulation strategies. The Chinese version was validated by Zhu et al. (2007) in Chinese university students, with confirmatory factor analysis supporting the original nine-factor structure.

The CERQ-C includes nine dimensions, each measured by four items: self-blame (SB; 4 items), acceptance (ACC; 4 items), rumination (RUM; 4 items), positive refocusing (PRF; 4 items), refocus on planning (RPL; 4 items), positive reappraisal (PRA; 4 items), putting into perspective (PIP; 4 items), catastrophizing (CAT; 4 items), and other-blame (OB; 4 items). Items are rated on a five-point Likert scale ranging from 1 = almost never to 5 = almost always. Dimension scores were calculated by averaging the four items within each dimension, with higher scores indicating more frequent use of the corresponding cognitive emotion regulation strategy.

For the CB-SEM model, the nine CERQ-C dimensions were organized into two theoretically meaningful latent variables. Adaptive cognitive emotion regulation (ACER) was indicated by ACC, PRF, RPL, PRA, and PIP. Maladaptive cognitive emotion regulation (MCER) was indicated by SB, RUM, CAT, and OB. Higher ACER scores indicate more frequent use of adaptive strategies, whereas higher MCER scores indicate more frequent use of maladaptive strategies. Representative item content included accepting what happened for ACC, thinking about pleasant things for PRF, planning what to do next for RPL, finding positive meaning in the situation for PRA, putting the event into a broader perspective for PIP, blaming oneself for SB, repeatedly thinking about one’s feelings for RUM, seeing the situation as terrible for CAT, and blaming others for OB.

#### 2.2.5 Temporal scope of measurement

The temporal scope of the measurements should be noted. PFLOW referred to students’ recalled experience during a recent piano performance, recital, examination performance, or public performance. By contrast, mindfulness, self-control, and cognitive emotion regulation were assessed as general self-reported tendencies rather than as momentary states recorded during performance. Therefore, the measures do not capture real-time regulatory processes during piano performance. The analyses examine whether general psychological regulatory tendencies are associated with retrospectively reported piano performance flow experience.

### 2.3 Analytical strategy

This study adopted a two-stage analytical strategy. First, structural equation modeling (SEM) was used to examine the net-association relationships among mindfulness, self-control, cognitive emotion regulation, and piano performance flow experience. Second, fuzzy-set qualitative comparative analysis (fsQCA) was used to identify configurations of psychological regulatory conditions associated with high and non-high piano performance flow experience. The combination of SEM and fsQCA was appropriate because SEM estimates average statistical relationships among variables, whereas fsQCA examines set-theoretic sufficiency, equifinality, and configurational asymmetry (Fiss, 2011; Pappas and Woodside, 2021; Ragin, 2008). In the present cross-sectional design, both SEM and fsQCA were used to examine association patterns rather than to establish causal or temporal ordering. Descriptive statistics, reliability analysis, correlations, and hierarchical regression were conducted using IBM SPSS Statistics 29.0; confirmatory factor analysis, structural equation modeling, and bootstrap indirect-association estimation were performed using AMOS 26.0; and configurational analyses were conducted using fsQCA 3.0.

#### 2.3.1 Structural equation modeling

Before testing the structural model, the data were screened for response quality and completeness. Because all substantive questionnaire items were mandatory in the online survey system, there were no item-level missing values. Responses that failed the attention-check item, showed extremely short completion time, displayed invariant or mechanically repeated response patterns, or failed to meet the inclusion criteria were removed before analysis. Descriptive statistics and Pearson correlations were then calculated for the main study variables.

The measurement properties of the reflective constructs were evaluated before the structural paths were tested. Because the study used multidimensional scales, measurement quality was assessed at two levels. First, item-level confirmatory factor analyses were conducted for the first-order dimensions or subdimensions. Second, dimension-level indicators were used to represent the higher-order constructs in the main SEM model. This approach reduced model complexity while retaining the theoretical multidimensionality of the constructs. It also allowed the piano-performance-adapted WOLF dimensions to be evaluated before ABS, ENJ, and IM were used as indicators of the higher-order PFLOW construct.

Internal consistency reliability was assessed using McDonald’s omega. Values of 0.70 or above were considered acceptable for research purposes (McDonald, 1999; Nunnally and Bernstein, 1994). Convergent validity was evaluated using standardized factor loadings, composite reliability (CR), and average variance extracted (AVE). Standardized factor loadings of 0.50 or above were considered acceptable, with values above 0.70 indicating stronger indicator reliability. CR values of 0.70 or above and AVE values of 0.50 or above were used as criteria for satisfactory convergent validity (Fornell and Larcker, 1981; Hair et al., 2019). Discriminant validity was examined using the Fornell–Larcker criterion, according to which the square root of AVE for each construct should exceed its correlations with other constructs (Fornell and Larcker, 1981).

Because all core variables were measured using self-report questionnaires, common method bias was assessed using both procedural and statistical approaches. Procedurally, the questionnaire emphasized anonymity, confidentiality, voluntary participation, and the absence of right or wrong answers. Different constructs were presented in separate sections, and an attention-check item was included to identify inattentive responding. Statistically, Harman’s single-factor exploratory factor analysis and confirmatory factor analysis were used to examine whether a single general factor or a latent common method factor could account for the covariance among the indicators. This combination of procedural and statistical remedies follows recommendations for addressing common method bias in behavioral research (Podsakoff et al., 2003).

The structural model was estimated using covariance-based SEM. Model fit was evaluated using multiple indices rather than relying only on the chi-square statistic, because chi-square is sensitive to sample size. The following indices were used: chi-square divided by degrees of freedom (χ^2^/df), comparative fit index (CFI), Tucker–Lewis index (TLI), standardized root mean square residual (SRMR), and root mean square error of approximation (RMSEA) with 90% confidence interval. In general, CFI and TLI values of 0.90 or higher indicate acceptable fit, and values of 0.95 or higher indicate good fit; SRMR values below 0.08 and RMSEA values below 0.08 indicate acceptable fit, with values around 0.06 or lower indicating good fit (Kline, 2023; Hu and Bentler, 1999).

The structural model was theory-specified but cross-sectional. Mindfulness was modeled as an exogenous predictor, self-control and cognitive emotion regulation as theoretically intermediate correlates, and piano performance flow experience as the criterion variable. Direct paths were specified from mindfulness to self-control and piano performance flow experience; from self-control to adaptive cognitive emotion regulation, maladaptive cognitive emotion regulation, and piano performance flow experience; and from both adaptive and maladaptive cognitive emotion regulation to piano performance flow experience. The arrows in the SEM represent theory-informed statistical associations and should not be interpreted as temporal or causal paths. Gender, grade, piano learning experience, weekly piano practice time, and recent piano performance context were included as control variables.

Indirect associations were estimated using a bootstrap procedure with 2,000 resamples. The bootstrap method was selected because it does not assume a normal sampling distribution of indirect estimates and is widely recommended for estimating confidence intervals around indirect associations (Hayes, 2018; Preacher and Hayes, 2008). Bootstrap confidence intervals were used to estimate the precision of indirect associations. This procedure was not used to infer causal mediation or temporal ordering. An indirect association was considered statistically significant when the 95% bootstrap confidence interval did not include zero. The analysis estimated the direct association between mindfulness and piano performance flow experience, the specific indirect associations through self-control and cognitive emotion regulation, the total indirect association, and the total association.

As a robustness check for the SEM findings, hierarchical regression analysis was conducted with piano performance flow experience as the dependent variable. In the first step, demographic and piano-related control variables were entered. In the second step, the four core theoretical predictors—mindfulness, self-control, adaptive cognitive emotion regulation, and maladaptive cognitive emotion regulation—were added. The change in explained variance (Δ*R*^2^) was used to evaluate whether the core theoretical predictors provided additional explanatory power beyond the control variables. This regression analysis was used as a complementary robustness check of the main association pattern rather than as an alternative causal or mediation model.

#### 2.3.2 Fuzzy-set qualitative comparative analysis

fsQCA was conducted to complement the SEM analysis by examining configurational profiles of psychological regulatory conditions. Unlike SEM, which estimates the independent net association between variables, fsQCA treats cases as configurations of conditions and identifies combinations that are sufficient for membership in an outcome set. This approach is suitable for examining equifinality and configurational asymmetry, especially when different combinations of conditions may be associated with the same outcome (Fiss, 2011; Ragin, 2008; Schneider and Wagemann, 2012). In the present cross-sectional design, fsQCA solutions are interpreted as set-theoretic association patterns rather than causal recipes.

The fsQCA analysis included four conditions, sometimes referred to in fsQCA terminology as causal conditions: mindfulness, self-control, adaptive cognitive emotion regulation, and maladaptive cognitive emotion regulation. Piano performance flow experience was used as the outcome set. Both high piano performance flow experience and non-high piano performance flow experience were analyzed to examine configurational asymmetry. In fsQCA, the configurations associated with membership in an outcome set are not assumed to be the simple inverse of the configurations associated with non-membership in that set (Ragin, 2008; Schneider and Wagemann, 2012).

Before the set-theoretic analysis, all variables were transformed into fuzzy-set membership scores using direct calibration. Direct calibration requires three qualitative anchors: full membership, crossover point, and full non-membership (Ragin, 2008). In this study, the calibration anchors were specified as follows: MIND was calibrated using 4.618, 3.646, and 2.475; SC was calibrated using 4.667, 3.617, and 2.400; ACER was calibrated using 4.550, 3.500, and 2.200; MCER was calibrated using 3.688, 2.500, and 1.438; and PFLOW was calibrated using 4.617, 3.567, and 2.250. The calibrated scores ranged from 0 to 1, where higher values indicated stronger membership in the corresponding set.

The fsQCA procedure consisted of necessity analysis, truth-table construction, sufficiency analysis, and robustness testing. Necessity analysis was first conducted to determine whether any single condition was necessary for high or non-high piano performance flow experience. A condition was considered necessary only when its consistency reached or exceeded 0.90, a commonly used threshold in set-theoretic research (Ragin, 2008; Schneider and Wagemann, 2012).

After necessity analysis, a truth table was constructed using the four conditions. With four conditions, the truth table contained 16 logically possible configurations. A case-frequency threshold was applied to avoid interpreting configurations with very limited empirical representation. For the main analysis, the minimum case-frequency cutoff was set at 33 cases. In addition, consistency and proportional reduction in inconsistency (PRI) were used to assess whether each truth-table row was sufficiently associated with the outcome set. Consistency reflects the degree to which cases sharing a configuration also belong to the outcome set, whereas PRI helps reduce simultaneous subset relations with both the presence and absence of the outcome (Ragin, 2008; Pappas and Woodside, 2021). Following common fsQCA practice, a consistency threshold of 0.80 or higher was used as the baseline criterion for sufficiency, and a PRI threshold of 0.70 was used in the main analysis to reduce contradictory configurations (Pappas and Woodside, 2021; Schneider and Wagemann, 2012).

The intermediate solution was used as the primary basis for interpretation because it balances empirical complexity and theoretical plausibility by incorporating theoretically informed expectations. The complex, parsimonious, and intermediate solutions were compared to determine the role of each condition. When a condition appeared in both the parsimonious and intermediate solutions, it was interpreted as a core condition; when it appeared only in the intermediate solution, it was interpreted as a peripheral condition (Fiss, 2011). Consistency, raw coverage, unique coverage, solution consistency, and solution coverage were reported for the final configurations. Raw coverage indicates how much of the outcome set is covered by a given configuration, unique coverage indicates the proportion of the outcome set covered only by that configuration, solution coverage indicates the overall empirical relevance of the solution, and solution consistency indicates the degree to which the overall solution is a subset of the outcome set (Ragin, 2008; Schneider and Wagemann, 2012).

Robustness checks were conducted to assess the stability of the configurational findings. Following recommendations that QCA results should be examined under alternative calibration and threshold choices, the fsQCA analysis was rerun under four alternative specifications (Greckhamer et al., 2018; Skaaning, 2011). First, the case-frequency threshold was adjusted to 1%, 1.5%, and 2% of the sample size. Second, the PRI threshold was lowered from 0.70 to 0.60. Third, the consistency threshold was increased from 0.80 to 0.85. Fourth, the calibration anchors were changed to the 75th percentile for full membership, the 50th percentile for the crossover point, and the 25th percentile for full non-membership. The robustness of the findings was evaluated by comparing whether the core configurational patterns remained substantively similar across these alternative specifications.

## 3 Results

### 3.1 Preliminary analyses

#### 3.1.1 Descriptive statistics and CFA-based measurement evidence

The measurement properties of the reflective constructs were first examined before testing the structural model. Because the study used multidimensional scales, item-level measurement quality was evaluated for each first-order dimension, and dimension-level indicators were then used to represent the higher-order constructs in the main model. Overall, the first-order dimensions showed satisfactory reliability and convergent validity, supporting their use as indicators of the corresponding higher-order constructs.

Because PFLOW was measured using a piano-performance-adapted WOLF, the internal structure of this adapted measure was examined before structural testing. A second-order CFA model was specified in which the 13 adapted WOLF items loaded onto three first-order factors—absorption, enjoyment, and intrinsic motivation—and these three first-order factors loaded onto the higher-order PFLOW construct. The model showed good fit to the data, χ^2^(62) = 164.552, *p* < 0.001, CFI = 0.988, TLI = 0.985, RMSEA = 0.033, 90% CI [0.027, 0.040], and SRMR = 0.021. The standardized second-order factor loadings from PFLOW to the three first-order factors ranged from 0.728 to 0.755, and the standardized first-order item loadings ranged from 0.719 to 0.810; all loadings were statistically significant, *p* < 0.001. PFLOW also showed satisfactory reliability and convergent validity, with McDonald’s *ω* = 0.908, CR = 0.784, and AVE = 0.548. These results support the use of ABS, ENJ, and IM as indicators of the higher-order PFLOW construct in the SEM.

Table 2 reports the descriptive statistics and measurement quality of the five higher-order constructs. McDonald’s omega values ranged from 0.908 to 0.958, indicating strong internal consistency. Composite reliability values ranged from 0.784 to 0.883, all exceeding the recommended threshold of 0.70. The standardized factor loadings ranged from 0.728 to 0.800, and all AVE values were above 0.50. These results indicate that mindfulness, self-control, adaptive cognitive emotion regulation, maladaptive cognitive emotion regulation, and piano performance flow experience had acceptable reliability and convergent validity.

**Table 2:** Descriptive statistics, internal consistency reliability, and CFA-based convergent validity of study variables.

Variable	*M*	SD	McDonald’s ω	Std. factor loading	CR	AVE
MIND	3.605	0.691	0.958	0.729–0.775	0.863	0.558
SC	3.584	0.722	0.934	0.758–0.786	0.883	0.600
ACER	3.435	0.744	0.935	0.745–0.789	0.874	0.582
MCER	2.508	0.732	0.918	0.744–0.800	0.856	0.598
PFLOW	3.503	0.768	0.908	0.728–0.755	0.784	0.548

M = mean; SD = standard deviation; ω = McDonald’s omega; CR = composite reliability; AVE = average variance extracted. Std. factor loading = standardized factor-loading range obtained from confirmatory factor analysis. MIND = mindfulness; SC = self-control; ACER = adaptive cognitive emotion regulation; MCER = maladaptive cognitive emotion regulation; PFLOW = piano performance flow experience. For PFLOW, the standardized factor-loading range refers to the second-order loadings from PFLOW to ABS, ENJ, and IM.

Table 3 presents the correlations among the study variables and the square roots of AVE. The correlation results were consistent with the proposed theoretical model. MIND, SC, and ACER were positively correlated with PFLOW, whereas MCER was negatively correlated with PFLOW. In addition, MIND and SC were positively associated with ACER and negatively associated with MCER. These findings provide preliminary support for the expected relationships among psychological regulatory resources and piano performance flow experience. Discriminant validity was assessed using the Fornell–Larcker criterion. The square root of AVE for each construct, shown on the diagonal of Table 3, was greater than its correlations with the other constructs. Therefore, the five constructs demonstrated adequate discriminant validity.

**Table 3:** Correlations and discriminant validity of the study variables.

**Variable**	**MIND**	**SC**	**ACER**	**MCER**	**PFLOW**
MIND	**0.747**				
SC	0.378^***^	**0.775**			
ACER	0.441^***^	0.629^***^	**0.763**		
MCER	−0.399^***^	−0.548^***^	−0.581^***^	**0.773**	
PFLOW	0.509^***^	0.511^***^	0.540^***^	−0.582^***^	**0.740**

Values on the diagonal in bold indicate the square roots of AVE. Discriminant validity is supported when the square root of AVE for each construct exceeds its correlations with other constructs. MIND = mindfulness; SC = self-control; ACER = adaptive cognitive emotion regulation; MCER = maladaptive cognitive emotion regulation; PFLOW = piano performance flow experience. *p < 0.05, **p < 0.01, ***p < 0.001.

#### 3.1.2 Common method bias assessment

Because all variables were measured using self-report questionnaires, several procedural and statistical remedies were adopted to reduce and assess the potential influence of common method bias. During questionnaire administration, participation was voluntary and anonymous, and respondents were informed that there were no right or wrong answers. The questionnaire emphasized confidentiality and encouraged participants to answer according to their actual experiences. Different constructs were presented in separate sections with clear instructions, and an attention-check item was included to identify inattentive responses. In addition, responses with extremely short completion time, invariant response patterns, failed attention checks, or other data-quality problems were removed before analysis.

After these procedural controls, common method bias was statistically assessed using Harman’s single-factor test and confirmatory factor analysis. First, an exploratory factor analysis was conducted with all measurement indicators entered simultaneously. The results extracted 22 factors with eigenvalues greater than 1, explaining 66.304% of the total variance. The first factor accounted for 24.379% of the variance, which was below the commonly used 40% threshold. This result indicates that a single general factor did not dominate the covariance among the observed variables.

A series of confirmatory factor analyses was then conducted to further examine common method bias. As shown in Table 4, the one-factor model showed poor fit to the data, χ^2^/df = 15.842, CFI = 0.764, TLI = 0.740, SRMR = 0.079, and RMSEA = 0.100. By contrast, the five-factor theoretical model showed good fit, χ^2^/df = 2.821, CFI = 0.972, TLI = 0.968, SRMR = 0.036, and RMSEA = 0.035. After adding a latent common method factor, the six-factor model did not show meaningful improvement over the five-factor model, with CFI, TLI, SRMR, and RMSEA remaining unchanged. These results do not eliminate the possibility of common method variance, but they indicate that a single general factor did not dominate the covariance structure.

**Table 4:** Model fit indices from confirmatory factor analyses for common method bias assessment.

Model	χ ^2^	df	χ ^2^/df	CFI	TLI	SRMR	RMSEA (90%CI)
One-factor model	3311.019	209	15.842	0.764	0.740	0.079	0.100 (0.097, 0.103)
Five-factor model	561.445	199	2.821	0.972	0.968	0.036	0.035 (0.032, 0.039)
Six-factor model	561.445	198	2.836	0.972	0.968	0.036	0.035 (0.032, 0.039)

The one-factor model specifies all indicators loading onto a single latent factor. The five-factor theoretical model specifies MIND, SC, ACER, MCER, and PFLOW as separate latent constructs. The six-factor model adds a latent common method factor in addition to the five theoretical constructs. CFI = comparative fit index; TLI = Tucker–Lewis index; SRMR = standardized root mean square residual; RMSEA = root mean square error of approximation; CI = confidence interval.

### 3.2 Structural equation modeling results

#### 3.2.1 Model fit and cross-sectional path estimates

The structural equation model was estimated to examine the direct associations among mindfulness, self-control, cognitive emotion regulation, and piano performance flow experience. The model showed acceptable fit to the data: χ^2^/df = 3.630, CFI = 0.960, TLI = 0.954, SRMR = 0.048, and RMSEA = 0.042, 90% CI [0.039, 0.046]. These results indicate that the proposed structural model adequately fitted the observed data.

**Figure 1:**
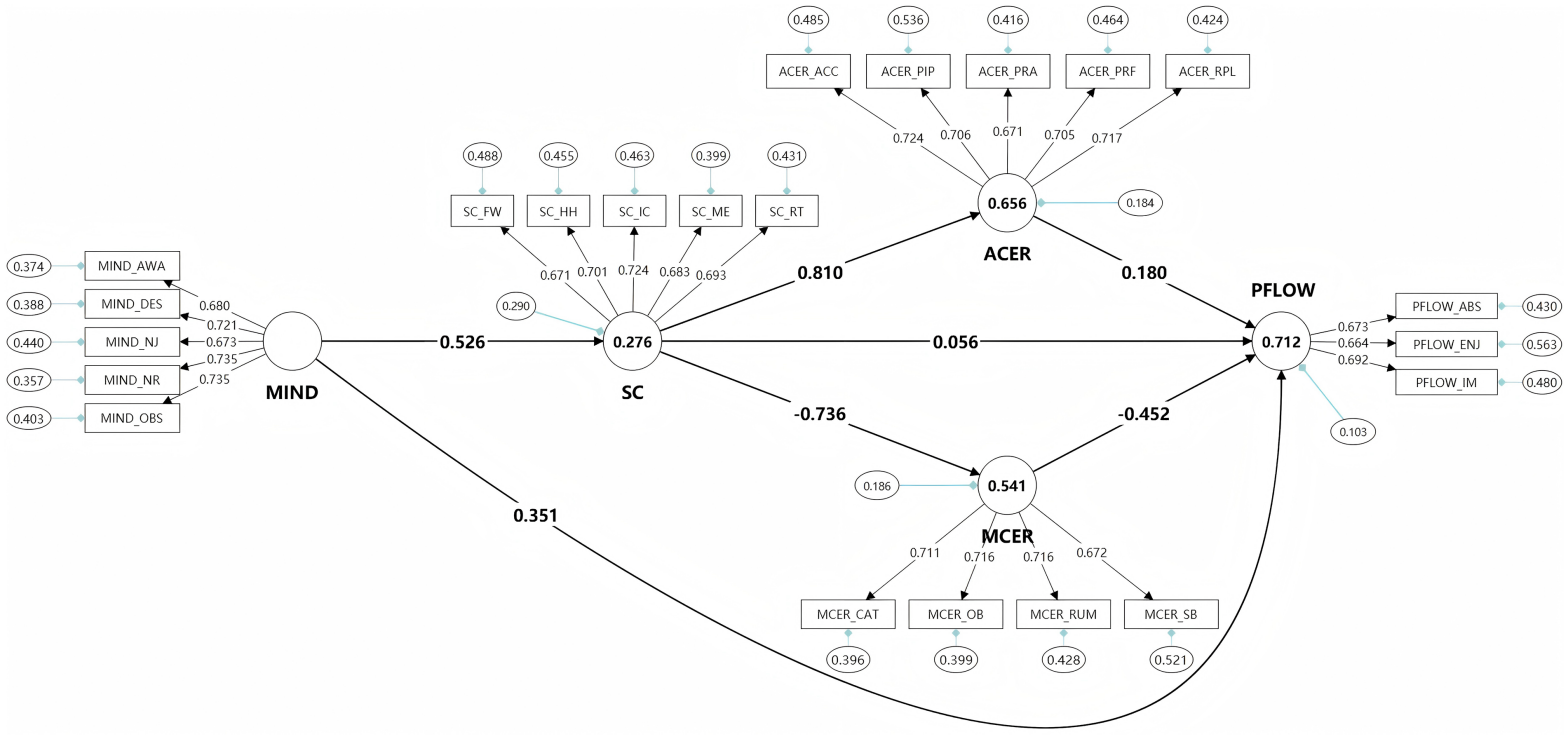
Standardized path coefficients of the cross-sectional structural equation model. Arrows indicate theory-informed statistical associations and should not be interpreted as temporal or causal paths.

As shown in Table 5 and Figure 1, mindfulness was positively associated with self-control (*β* = 0.526, *p* < 0.001). Self-control was positively associated with adaptive cognitive emotion regulation (*β* = 0.810, *p* < 0.001) and negatively associated with maladaptive cognitive emotion regulation (*β* = −0.736, *p* < 0.001). These results suggest that students with higher mindfulness tended to report stronger self-control, and those with stronger self-control tended to report more adaptive and less maladaptive cognitive emotion regulation.

**Table 5:** Standardized path estimates of the cross-sectional SEM model.

Path	*β*	SE	C.R.
MIND → SC	0.526^***^	0.039	15.061
SC → ACER	0.810^***^	0.046	20.582
SC → MCER	−0.736^***^	0.039	−18.900
ACER → PFLOW	0.180^**^	0.044	3.335
MCER → PFLOW	−0.452^***^	0.045	−9.515
MIND → PFLOW	0.351^***^	0.036	10.220
SC → PFLOW	0.056	0.060	0.871

β = standardized path coefficient; SE = standard error. Paths represent theory-informed cross-sectional associations and should not be interpreted as temporal or causal effects. MIND = mindfulness; SC = self-control; ACER = adaptive cognitive emotion regulation; MCER = maladaptive cognitive emotion regulation; PFLOW = piano performance flow experience. Control variables were included but are not shown in the table. *p < 0.05, **p < 0.01, ***p < 0.001.

The two types of cognitive emotion regulation showed opposite associations with piano performance flow experience. Adaptive cognitive emotion regulation was positively associated with piano performance flow experience (*β* = 0.180, *p* < 0.01), whereas maladaptive cognitive emotion regulation was negatively associated with piano performance flow experience (*β* = −0.452, *p* < 0.001). In addition, mindfulness had a positive direct association with piano performance flow experience (*β* = 0.351, *p* < 0.001). By contrast, the direct path from self-control to piano performance flow experience was not statistically significant (*β* = 0.056, *p* > 0.05) after the other variables were included in the model.

The model explained 27.6% of the variance in self-control, 65.6% of the variance in adaptive cognitive emotion regulation, 54.1% of the variance in maladaptive cognitive emotion regulation, and 71.2% of the variance in piano performance flow experience. These results provided the basis for estimating indirect associations using bootstrap confidence intervals.

#### 3.2.2 Bootstrap estimates of indirect associations

Bootstrap analysis was conducted to estimate the indirect associations between mindfulness and piano performance flow experience through self-control and cognitive emotion regulation. A bootstrap procedure with 2,000 resamples was used, and an indirect association was considered statistically significant when the 95% bootstrap confidence interval did not include zero.

As shown in Table 6, the total association between mindfulness and piano performance flow experience was significant, *β* = 0.632, 95% CI [0.579, 0.682]. The direct association remained significant after self-control and cognitive emotion regulation were included as intermediate correlates, *β* = 0.351, 95% CI [0.288, 0.413], representing 55.54% of the total standardized association. The total indirect association was also significant, *β* = 0.281, 95% CI [0.243, 0.324], representing 44.46% of the total standardized association. These results indicate that mindfulness was associated with piano performance flow experience both directly and indirectly through psychological regulatory association patterns.

**Table 6:** Bootstrap estimates of total, direct, and indirect associations.

Association type	Path	β	Boot SE	Boot LLCI	Boot ULCI	Ratio %
Direct association	MIND → PFLOW	0.351	0.033	0.288	0.413	55.54
Indirect association	MIND → SC → PFLOW	0.029	0.036	−0.044	0.096	4.59
Indirect association	MIND → SC → ACER → PFLOW	0.077	0.026	0.030	0.131	12.18
Indirect association	MIND → SC → MCER → PFLOW	0.175	0.021	0.137	0.216	27.69
Total indirect association	All indirect paths	0.281	0.020	0.243	0.324	44.46
Total association	MIND → PFLOW	0.632	0.026	0.579	0.682	100.00

β = standardized estimate; Boot SE = bootstrap standard error; Boot LLCI = lower limit of the 95% bootstrap confidence interval; Boot ULCI = upper limit of the 95% bootstrap confidence interval. Bootstrap sample size = 2,000. An indirect association is statistically significant when the 95% confidence interval does not include zero. Because the data are cross-sectional, these estimates are interpreted as indirect associations rather than causal mediation effects. MIND = mindfulness; SC = self-control; ACER = adaptive cognitive emotion regulation; MCER = maladaptive cognitive emotion regulation; PFLOW = piano performance flow experience.

Among the specific indirect associations, the indirect association through self-control alone was not significant, *β* = 0.029, 95% CI [−0.044, 0.096]. In contrast, the indirect association through self-control and adaptive cognitive emotion regulation was significant, *β* = 0.077, 95% CI [0.030, 0.131], representing 12.18% of the total standardized association. The indirect association through self-control and maladaptive cognitive emotion regulation was also significant, *β* = 0.175, 95% CI [0.137, 0.216], representing 27.69% of the total standardized association.

The largest indirect association was observed for the statistical route from mindfulness to self-control, then to maladaptive cognitive emotion regulation, and finally to piano performance flow experience. Because self-control was negatively associated with maladaptive cognitive emotion regulation and maladaptive cognitive emotion regulation was negatively associated with piano performance flow experience, this indirect association was positive overall. This pattern is consistent with the possibility that mindfulness is linked to stronger recalled PFLOW through its associations with self-control and lower maladaptive cognitive regulation.

#### 3.2.3 Robustness test

A hierarchical regression analysis was conducted as a robustness test to examine whether the core theoretical predictors remained associated with piano performance flow experience after controlling for demographic and piano-related variables. Model 1 included gender, grade, piano learning experience, weekly piano practice time, and recent piano performance context as control variables. Model 2 added mindfulness, self-control, adaptive cognitive emotion regulation, and maladaptive cognitive emotion regulation.

As shown in Table 7, Model 1 explained 7.2% of the variance in piano performance flow experience, *R*^2^ = 0.072, adjusted *R*^2^ = 0.069. Gender and grade were significant predictors in this control-variable-only model. After the four core theoretical predictors were added in Model 2, the explained variance increased substantially, *R*^2^ = 0.478, adjusted *R*^2^ = 0.475, Δ*R*^2^ = 0.405. This indicates that mindfulness, self-control, adaptive cognitive emotion regulation, and maladaptive cognitive emotion regulation together provided substantial additional explanatory power beyond the control variables.

**Table 7:** Robustness test of core predictors on piano performance flow experience: Hierarchical regression results.

Variable	Model 1	Model 2
Gender	0.102***	0.033
Grade	0.244***	0.077***
Piano learning experience	−0.032	−0.013
Weekly piano practice time	−0.018	−0.002
Recent piano performance context	−0.024	0.014
MIND		0.253***
SC		0.145***
ACER		0.139***
MCER		−0.304***
*R* ^2^	0.072	0.478
Adjusted *R*^2^	0.069	0.475
Δ*R*^2^	0.072	0.405
*F*	*F* (5, 1,470) = 22.978, *p* < 0.001	*F* (9, 1,466) = 148.998, *p* < 0.001

The dependent variable is PFLOW. Model 1 includes only control variables. Model 2 adds the core theoretical predictors: MIND, SC, ACER, and MCER. Standardized regression coefficients are reported. MIND = mindfulness; SC = self-control; ACER = adaptive cognitive emotion regulation; MCER = maladaptive cognitive emotion regulation; PFLOW = piano performance flow experience. *p < 0.05, **p < 0.01, ***p < 0.001.

In Model 2, mindfulness, self-control, and adaptive cognitive emotion regulation were positively associated with piano performance flow experience, whereas maladaptive cognitive emotion regulation was negatively associated with piano performance flow experience. Among the core predictors, maladaptive cognitive emotion regulation showed the strongest absolute association with piano performance flow experience. These results were broadly consistent with the structural equation modeling results and supported the robustness of the main association pattern. Because the regression model did not test the same indirect-association structure as the SEM model, it was used only as a complementary robustness check rather than as an alternative indirect-association model.

The control-variable pattern also deserves attention. In Model 1, gender and grade were significant predictors of PFLOW, and the control variables explained 7.2% of the variance. After the four psychological variables were added in Model 2, the gender coefficient became nonsignificant, whereas the grade coefficient was attenuated but remained statistically significant. This pattern suggests that gender differences in recalled PFLOW may overlap with differences in psychological regulatory resources, whereas educational stage may retain a small independent association with PFLOW.

### 3.3 Fuzzy-set qualitative comparative analysis results

#### 3.3.1 Necessity condition analysis

Before the necessity and sufficiency analyses, the study variables were transformed into fuzzy-set membership scores using direct calibration. Three anchors were specified for each variable: full membership, crossover point, and full non-membership. The calibration anchors were as follows: MIND was calibrated using 4.618, 3.646, and 2.475; SC was calibrated using 4.667, 3.617, and 2.400; ACER was calibrated using 4.550, 3.500, and 2.200; MCER was calibrated using 3.688, 2.500, and 1.438; and PFLOW was calibrated using 4.617, 3.567, and 2.250. After calibration, MIND, SC, ACER, and MCER were used as conditions, and PFLOW was used as the outcome set.

A necessity condition analysis was then conducted for both high and non-high piano performance flow experience. In fsQCA, a condition is generally regarded as necessary when its consistency reaches or exceeds 0.90. As shown in Table 8, none of the single conditions reached this threshold for either high PFLOW or non-high PFLOW. For high PFLOW, the highest consistency value was observed for ~MCER, with a consistency of 0.781 and a coverage of 0.773. MIND, SC, and ACER also showed relatively high consistency values, but all remained below the 0.90 criterion. Therefore, although low maladaptive cognitive emotion regulation, mindfulness, self-control, and adaptive cognitive emotion regulation were relevant to high piano performance flow experience, no single condition alone was necessary for membership in high PFLOW. For non-high PFLOW, relatively high consistency values were found for ~ACER, MCER, ~SC, and ~MIND, ranging from 0.751 to 0.767. However, these values also did not meet the necessity threshold. This indicates that non-high piano performance flow experience was not characterized by any single necessary condition alone. Overall, the necessity analysis supports the use of a configurational perspective.

**Table 8:** Necessary condition analysis for high and non-high piano performance flow experience.

Condition	High PFLOW consistency	High PFLOW coverage	Non-high PFLOW consistency	Non-high PFLOW coverage
MIND	0.758	0.758	0.502	0.488
~MIND	0.487	0.501	0.751	0.751
SC	0.758	0.759	0.512	0.499
~SC	0.499	0.513	0.753	0.752
ACER	0.763	0.771	0.498	0.490
~ACER	0.495	0.503	0.767	0.759
MCER	0.472	0.491	0.764	0.773
~MCER	0.781	0.773	0.496	0.478

“~” denotes the absence or low level of a given condition. A condition is generally considered necessary when consistency reaches or exceeds 0.90. MIND = mindfulness; SC = self-control; ACER = adaptive cognitive emotion regulation; MCER = maladaptive cognitive emotion regulation; PFLOW = piano performance flow experience.

#### 3.3.2 Conditional configuration analysis

Because no single condition constituted a necessary condition, a sufficiency analysis was conducted to identify configurations associated with high and non-high piano performance flow experience. In line with the cross-sectional design, the fsQCA solutions are interpreted as configurations associated with set membership rather than causal recipes. The intermediate solution was used as the primary basis for interpretation. Because the complex, parsimonious, and intermediate solutions were identical, all conditions reported in Table 9 were treated as core conditions.

**Table 9:** Configurational solutions associated with high and non-high piano performance flow experience.

Conditions	High PFLOW S1	High PFLOW S2	Non-high PFLOW NS1
MIND	●	●	⨂
SC	●		
ACER		●	⨂
MCER	⨂	⨂	●
Consistency	0.904	0.898	0.902
Raw coverage	0.541	0.551	0.555
Unique coverage	0.043	0.054	0.555
Solution consistency	0.888		0.902
Solution coverage	0.594		0.555

● indicates the presence of a core condition; ⨂ indicates the absence of a core condition; blank cells indicate that the condition may be either present or absent. Because the complex, parsimonious, and intermediate solutions were identical, no peripheral conditions were distinguished. MIND = mindfulness; SC = self-control; ACER = adaptive cognitive emotion regulation; MCER = maladaptive cognitive emotion regulation; PFLOW = piano performance flow experience.

As shown in Table 9, two configurations were identified for high PFLOW. The first configuration, S1, consisted of high mindfulness, high self-control, and low maladaptive cognitive emotion regulation. This configuration showed a consistency of 0.904, a raw coverage of 0.541, and a unique coverage of 0.043. This configuration was associated with stronger membership in high piano performance flow when strong mindfulness and self-control were combined with a low level of maladaptive cognitive emotion regulation.

The second configuration, S2, consisted of high mindfulness, high adaptive cognitive emotion regulation, and low maladaptive cognitive emotion regulation. This configuration showed a consistency of 0.898, a raw coverage of 0.551, and a unique coverage of 0.054. High piano performance flow membership was also characterized by the combination of mindfulness, adaptive cognitive emotion regulation, and low maladaptive cognitive emotion regulation, even when self-control was not a defining condition.

The overall solution consistency for high PFLOW was 0.888, and the overall solution coverage was 0.594. These findings indicate that the two configurations had acceptable consistency and covered a meaningful proportion of high-flow cases. Substantively, both high-PFLOW configurations shared two core conditions: high mindfulness and low maladaptive cognitive emotion regulation. However, they differed in the supporting regulatory condition: S1 emphasized self-control, whereas S2 emphasized adaptive cognitive emotion regulation. This pattern reflects equifinality, meaning that high piano performance flow experience may be characterized by more than one psychological regulatory profile.

For non-high PFLOW, one configuration was identified. NS1 consisted of low mindfulness, low adaptive cognitive emotion regulation, and high maladaptive cognitive emotion regulation. This configuration showed a consistency of 0.902, a raw coverage of 0.555, and a unique coverage of 0.555. This result suggests that non-high piano performance flow membership was characterized by the combination of low mindfulness, low adaptive cognitive emotion regulation, and high maladaptive cognitive emotion regulation.

The non-high PFLOW configuration was not a simple mirror image of the high-PFLOW configurations. In the high-PFLOW configurations, self-control or adaptive cognitive emotion regulation combined with mindfulness and low MCER to characterize membership in the high-PFLOW outcome set. In the non-high PFLOW configuration, however, the defining pattern was low mindfulness, low ACER, and high MCER, while self-control was not a defining condition. This finding reflects configurational asymmetry in the patterning of piano performance flow experience.

#### 3.3.3 Robustness test

Several robustness checks were conducted to evaluate the stability of the fsQCA results. Following recommended fsQCA practice, the analysis was rerun by adjusting key analytical settings that may influence configurational solutions. Specifically, alternative case-frequency thresholds corresponding to 1%, 1.5%, and 2% of the sample size were additionally tested. The PRI consistency threshold was lowered from 0.70 to 0.60, the overall consistency threshold was increased from 0.80 to 0.85, and the calibration anchors were changed to the 75th percentile for full membership, the 50th percentile for the crossover point, and the 25th percentile for full non-membership.

The robustness checks showed that the core configurational interpretation remained generally stable. For high PFLOW, mindfulness and low maladaptive cognitive emotion regulation consistently appeared as important elements in the configurations associated with high piano performance flow experience. Depending on the analytical specification, self-control or adaptive cognitive emotion regulation appeared as an additional supporting condition. For non-high PFLOW, the main pattern continued to involve low mindfulness, weak adaptive cognitive emotion regulation, and high maladaptive cognitive emotion regulation.

Some variation appeared in the number of configurations when the PRI threshold was lowered to 0.60. This is theoretically expected because a lower PRI threshold allows more truth-table rows to enter the minimization process. However, the additional configurations did not alter the core interpretation of the findings. Overall, the robustness analyses supported the interpretation that high piano performance flow was associated with the presence of positive psychological regulatory resources and low maladaptive cognitive emotion regulation, whereas non-high piano performance flow was associated with a regulatory risk profile. Therefore, the fsQCA findings were considered robust in terms of their core theoretical meaning, although some solution complexity varied across alternative analytical specifications.

## 4 Discussion

### 4.1 Psychological regulation and the fragile nature of piano performance flow

This study examined piano performance flow experience among art students at Chinese sport universities from a psychological regulatory perspective. The findings indicate that piano performance flow should not be understood only as a pleasant by-product of musical skill or practice experience. Rather, retrospectively reported piano performance flow was closely associated with students’ regulatory resources, especially mindfulness, self-control, adaptive cognitive emotion regulation, and maladaptive cognitive emotion regulation.

The findings should be interpreted within the cross-sectional and retrospective measurement design. PFLOW referred to recalled experience during a recent piano performance, whereas mindfulness, self-control, and cognitive emotion regulation were measured as general self-reported tendencies. Therefore, the results do not demonstrate moment-to-moment in-performance regulatory dynamics. Rather, they show that students’ general regulatory tendencies were associated with how they retrospectively reported piano performance flow.

This interpretation is important because piano performance is a fragile optimal experience. During performance, students must remain immersed in musical expression while monitoring sound, movement, memory, audience evaluation, and possible mistakes (Furuya et al., 2021). Under such conditions, flow may be disrupted not only by technical difficulty but also by intrusive self-evaluation, worry, rumination, and catastrophizing (Kenny, 2011). This is consistent with attentional control theory, which argues that anxiety and threat-related processing can weaken goal-directed attentional control and increase stimulus-driven attention (Eysenck et al., 2007). Although the present study did not directly measure music performance anxiety or momentary regulation during performance, the strong negative association between maladaptive cognitive emotion regulation and piano performance flow is consistent with the view that students’ cognitive responses to performance pressure are closely linked to whether they report stronger or weaker flow experience (Yao and Li, 2022). This interpretation is theoretically plausible, but it should not be read as direct evidence of regulation occurring during performance.

The results also help clarify the role of mindfulness in performance flow. Mindfulness was directly associated with piano performance flow and also appeared as a core condition in both high-flow fsQCA configurations. This pattern is compatible with the view that mindfulness provides an attentional basis for flow by supporting present-moment awareness and reducing automatic reactivity (Bishop et al., 2004). In performance settings, this may mean that students who report higher mindfulness are more likely to remain connected to the musical task rather than becoming absorbed by self-critical thoughts or anticipated mistakes. Previous sport psychology research has also linked mindfulness characteristics with flow dispositions and mental skills, suggesting that present-focused awareness is relevant to optimal performance states across embodied performance contexts (Kee and Wang, 2008; Martiny et al., 2024). In the present study, however, mindfulness was assessed as a general self-reported tendency, so the finding should be interpreted as an association between dispositional mindfulness and recalled flow rather than as evidence that mindfulness operated moment by moment during performance.

The necessity analysis showed that mindfulness alone was not a necessary condition for high piano performance flow. This is theoretically meaningful. It suggests that mindfulness should not be treated as a universal or isolated explanation of flow. In this study, mindfulness mattered most when it appeared in combination with other regulatory conditions, especially low maladaptive cognitive emotion regulation and either self-control or adaptive cognitive emotion regulation. This supports a more relational view of psychological regulation: present-moment awareness is important, but its relevance for flow may depend on how it is embedded in broader patterns of self-regulation and emotional appraisal.

### 4.2 Self-control as a regulatory background rather than a direct flow experience

A notable finding is that self-control was strongly associated with adaptive and maladaptive cognitive emotion regulation, but its direct path to piano performance flow was not statistically significant in the SEM model after other variables were included. This should not be interpreted as meaning that self-control is unimportant. Rather, it suggests that self-control may be more closely linked to the regulatory background of flow than to the immediate subjective experience of flow itself (Antonini Philippe et al., 2022; Moral-Bofill et al., 2022).

This distinction is theoretically useful. Flow is often experienced as smooth, absorbed, and intrinsically rewarding, whereas self-control is often described in terms of impulse regulation, goal maintenance, and resistance to distraction. In piano performance, too much deliberate control over already learned motor sequences may even interfere with fluent execution. Research on skilled performance under pressure has shown that excessive attention to proceduralized skills can undermine performance fluency (Beilock and Carr, 2001). Therefore, the non-significant direct path from self-control to flow in the SEM model may reflect the possibility that self-control is not the experiential core of flow, but a regulatory resource associated with practice habits, distraction management, and emotional responses surrounding performance. Because the present study did not measure self-control during actual performance, this explanation should be treated as an interpretation consistent with the observed associations rather than as direct evidence of in-performance control processes.

This interpretation is also compatible with contemporary self-control theory. Self-control is not only an effortful in-the-moment struggle against impulses; it may also involve earlier-stage strategies such as situation selection, situation modification, and attentional deployment (Duckworth et al., 2016). In the present study, self-control was strongly associated with higher adaptive cognitive emotion regulation and lower maladaptive cognitive emotion regulation. This pattern suggests that among art students in sport universities, self-control may be linked to how students organize their regulatory responses to performance demands rather than simply to whether they feel flow directly.

The hierarchical regression robustness test also showed that self-control remained positively associated with piano performance flow when entered alongside the other core predictors. This does not contradict the SEM result. The regression model estimated the unique association of composite-level predictors with PFLOW, whereas the SEM model partitioned the relationships among mindfulness, self-control, adaptive cognitive emotion regulation, and maladaptive cognitive emotion regulation within a structured association model. Taken together, these results suggest that self-control is relevant to piano performance flow, but its meaning is more nuanced than a simple direct association.

### 4.3 The central role of maladaptive cognitive emotion regulation

Across the SEM, bootstrap, and fsQCA results, maladaptive cognitive emotion regulation emerged as a particularly important construct. In the SEM model, MCER showed the strongest direct association with PFLOW in absolute value. In the bootstrap analysis, the indirect association involving self-control and MCER accounted for the largest proportion of the total standardized association. In the fsQCA results, low MCER appeared in both high-flow configurations, whereas high MCER appeared in the non-high-flow configuration.

This convergent pattern suggests that the absence of maladaptive cognitive responses may be especially relevant to piano performance flow. Strategies such as self-blame, rumination, catastrophizing, and other-blame can sustain negative cognitive focus after stressful events (Garnefski et al., 2001; Aldao et al., 2010). Such cognitive responses are difficult to reconcile with the attentional absorption and enjoyment that characterize flow. A broad meta-analysis of emotion-regulation strategies has shown that maladaptive strategies such as rumination and avoidance are more strongly associated with negative psychological outcomes than adaptive strategies are associated with positive outcomes (Aldao et al., 2010). Although the present study focused on piano performance flow rather than psychopathology, the pattern is conceptually similar: maladaptive cognitive regulation appears to be closely connected to a weaker optimal experience (Marin and Bhattacharya, 2013). Because the study did not assess students’ thoughts during a live performance, this interpretation should be understood as theoretically consistent with the observed association rather than as direct evidence of real-time cognitive disruption.

This finding also enriches the interpretation of adaptive cognitive emotion regulation. ACER was positively associated with PFLOW and appeared in one of the high-flow configurations, but the results suggest that adaptive strategies may be most meaningful when maladaptive strategies are low (Aldao et al., 2014; Aldao and Nolen-Hoeksema, 2012). In other words, positive reappraisal, planning, acceptance, and perspective-taking may support flow, but they do not simply replace the need to reduce rumination or catastrophizing. For piano students, the challenge may not only be to “think positively” after a mistake, but also to avoid becoming trapped in self-blame or catastrophic interpretation while performing or reflecting on performance.

This point has pedagogical relevance, but it should be interpreted cautiously. In piano education, teachers often respond to performance problems by giving more technical advice, more practice requirements, or more evaluative feedback. The present findings suggest that cognitive responses to pressure and mistakes deserve attention as possible targets for future research. Students who repeatedly interpret mistakes as personal failure or evidence of incompetence may have difficulty remaining immersed in performance, even when they possess sufficient technical preparation. However, this study did not test whether changing such cognitive responses would improve flow.

### 4.4 What SEM and fsQCA jointly reveal

The combined use of SEM and fsQCA adds an important layer to the interpretation. SEM showed the average associations among mindfulness, self-control, cognitive emotion regulation, and piano performance flow. fsQCA showed that high flow and non-high flow were linked to different combinations of conditions. These two forms of evidence answer different questions and should not be collapsed into a single linear explanation.

The SEM results indicate that mindfulness, adaptive cognitive emotion regulation, and maladaptive cognitive emotion regulation were directly associated with piano performance flow, and that mindfulness was also indirectly associated with flow through self-control and cognitive emotion regulation. This provides a net-association explanation: on average, students with higher mindfulness and adaptive regulation and lower maladaptive regulation tended to report stronger piano performance flow. Given the cross-sectional design, these indirect associations should not be interpreted as causal mediation effects.

The fsQCA results provide a configurational explanation. High PFLOW was associated with two configurations: one combining high mindfulness, high self-control, and low MCER; the other combining high mindfulness, high ACER, and low MCER. These configurations suggest that high flow does not require every positive regulatory resource to be simultaneously present. For some students, the relevant profile may involve mindfulness, self-control, and low maladaptive regulation. For others, the relevant profile may involve mindfulness, adaptive cognitive regulation, and low maladaptive regulation. This reflects equifinality, a core principle of set-theoretic approaches (Schneider and Wagemann, 2012).

The non-high PFLOW configuration further showed configurational asymmetry. Non-high flow was associated with low mindfulness, low adaptive cognitive emotion regulation, and high maladaptive cognitive emotion regulation. This was not a simple reversal of the high-flow configurations, because low self-control was not a defining condition in the non-high-flow configuration. This asymmetry is important for theory building. It suggests that the conditions linked to strong flow are not merely the inverse of those linked to weak flow. In practical terms, future research may examine whether helping students approach high flow requires building mindfulness and reducing maladaptive regulation, whereas identifying students at risk of non-high flow may require attention to the combination of low mindfulness, weak adaptive appraisal, and high maladaptive cognitive response (Hohnemann et al., 2024).

### 4.5 Demographic and educational-stage patterns

The control-variable results also provide useful context. Gender and grade were significantly associated with PFLOW in the control-variable-only model. After mindfulness, self-control, ACER, and MCER were entered, the gender coefficient became nonsignificant, whereas the grade coefficient was attenuated but remained statistically significant. This pattern suggests that gender differences in recalled PFLOW may partly overlap with psychological regulatory resources, whereas educational stage may retain a small independent association.

One possible explanation is that students in higher grades may have accumulated more piano-performance exposure, course-examination experience, or familiarity with evaluative settings. Such accumulated experience may make students more familiar with performance demands and may shape how they recall and evaluate flow-like experiences. However, because the study was cross-sectional and did not test gender- or grade-specific mechanisms, these interpretations should remain cautious. Future research should examine whether the association pattern differs by gender or educational stage using longitudinal, multi-group, or performance-based designs.

### 4.6 Theoretical implications

This study offers three theoretical implications. First, it contributes to flow research in music performance by showing that retrospectively reported piano performance flow is associated with a constellation of psychological regulatory resources. Previous research has often emphasized flow as a state of deep absorption and intrinsic enjoyment, but the present study highlights regulatory resources associated with that state. This helps move the discussion of piano performance flow beyond musical skill alone and toward a broader framework that includes attentional awareness, self-regulatory capacity, and cognitive emotional appraisal.

Second, the study refines the role of self-control in performance flow. Rather than treating self-control as a simple direct predictor of flow, the findings suggest that self-control is better understood as a regulatory resource associated with the organization of cognitive emotion regulation. This is consistent with a broader view of self-control as proactive regulation rather than only effortful suppression (Duckworth et al., 2016; de Ridder et al., 2012). In piano performance, self-control may be most relevant before and around the flow experience: in maintaining practice discipline, managing distractions, and shaping responses to pressure. This interpretation also avoids the misleading assumption that more conscious control during performance is always beneficial.

Third, the study contributes methodologically to performance psychology by integrating SEM and fsQCA. The SEM results provide a net-association explanation, whereas the fsQCA results reveal multiple configurations and configurational asymmetry. This integration is useful because optimal performance experiences are unlikely to be characterized by one isolated factor alone. They are better understood as patterns of conditions that may operate differently across individuals. By combining these approaches, the study provides a more nuanced cross-sectional account of piano performance flow among art students at Chinese sport universities.

### 4.7 Practical implications

Because the present study was cross-sectional, self-report, and non-interventional, the practical implications should be interpreted as candidate directions for future pedagogical and intervention research rather than as evidence that specific techniques improve piano performance flow. The findings point to possible psychological resources and regulatory profiles that may deserve further examination in piano education within sport-university contexts.

First, future intervention studies may examine whether brief attention-centering routines before performance are associated with stronger momentary flow. Such routines may include breath-based focusing, body scanning, or sound-focused listening (Hawkes, 2021; Iott, 2021). These activities should not be treated as techniques already validated by the present study. Rather, they represent theoretically plausible practices that could be tested in relation to performance-specific attention, tension regulation, and the ability to return to the musical line after small mistakes.

Second, structured post-error and post-performance reflection may be a theoretically plausible target for future pedagogical research. Since maladaptive cognitive emotion regulation was strongly associated with weaker recalled flow and appeared as a defining condition in the non-high-flow configuration, future studies could test whether guided reflection helps students distinguish between technical feedback, emotional reaction, and self-evaluative interpretation (Kruse-Weber and Parncutt, 2014). For example, after a class performance, students could be asked “What did I notice?,” “What can be adjusted next time?,” and “How can I return attention to the musical task?” Such prompts are aligned with adaptive strategies such as refocus on planning, positive reappraisal, and putting into perspective (McPherson, 2022), but their effects on flow require empirical testing.

Third, future research may examine whether self-control-related support is more effective when embedded in practice organization rather than framed as a demand for stronger willpower. Research on musical self-regulation emphasizes that learning an instrument requires students to become active participants in planning, monitoring, and evaluating their own practice (McPherson and Zimmerman, 2011). For art students at sport universities, possible intervention components may include goal setting, distraction management, staged performance simulation, and recovery strategies after mistakes. This direction is consistent with the present finding that self-control was closely linked to cognitive emotion regulation rather than functioning only as a direct correlate of flow in the SEM model.

Fourth, the fsQCA profiles may generate hypotheses about differentiated support, but they should not be treated as validated diagnostic or intervention profiles. Students who show strong self-control may benefit from approaches that examine maladaptive cognitive responses before and after performance (Easdale-Cheele et al., 2026). Students who show weaker self-control but stronger adaptive appraisal may still report high flow when mindfulness and low maladaptive regulation are present. Conversely, students showing low mindfulness, low adaptive regulation, and high maladaptive regulation may require broader psychological support rather than additional performance pressure alone (Biasutti and Philippe, 2023). These directions require experimental, longitudinal, or performance-based testing before they can be translated into evidence-based pedagogical prescriptions.

### 4.8 Limitations and future research

Several limitations should be considered. First, the study used a cross-sectional survey design. This limitation is central to the interpretation of the study. Although the SEM and bootstrap analyses estimated theoretically informed indirect associations, the data do not establish temporal order or causality. The study cannot determine whether mindfulness temporally preceded self-control, whether self-control preceded cognitive emotion regulation, or whether these variables changed before, during, or after the performance. Cross-sectional mediation models can produce biased estimates when interpreted as longitudinal processes (Maxwell and Cole, 2007; Maxwell et al., 2011). Therefore, the present findings should be interpreted as patterns of association rather than evidence that mindfulness, self-control, or cognitive emotion regulation causes piano performance flow. Future research should use longitudinal designs, experience sampling, or repeated performance assessments to examine how regulatory states and flow fluctuate before, during, and after piano performance.

Second, all main variables were measured using self-report questionnaires. The study adopted procedural controls and statistical tests for common method bias, but self-report designs cannot fully eliminate shared method variance or retrospective response bias. PFLOW was recalled rather than measured during or immediately after performance. Method-bias research emphasizes that procedural remedies and statistical checks are useful but cannot guarantee the absence of method effects (Podsakoff et al., 2003). Future studies could combine self-report flow measures with teacher ratings, behavioral indicators, physiological markers, or performance-based assessments. For example, momentary flow reports immediately after a performance could be compared with heart-rate variability, performance error patterns, or external evaluations of musical expressiveness.

Third, piano performance flow was measured using a piano-performance-adapted version of WOLF. The current-sample CFA-based evidence supported the reliability, convergent validity, and discriminant validity of the adapted PFLOW measure, and the second-order structure of absorption, enjoyment, and intrinsic motivation was supported in the present sample. However, the scale was originally developed for work-related flow, and the present study should not be interpreted as a full validation of a new music-specific flow instrument. The study also did not provide external convergent-validity evidence against FSS-2 or another music-specific flow measure (Jackson and Eklund, 2002). This adaptation is theoretically defensible because absorption, enjoyment, and intrinsic motivation are relevant to performance flow, but future studies should compare this measure with music-specific or performance-specific flow instruments. Such work would clarify whether piano performance flow is best represented by a three-component structure or by broader dimensions such as challenge–skill balance, clear goals, sense of control, and loss of self-consciousness.

Fourth, the sample consisted of art students from Chinese sport universities. This group was central to the purpose of the study, but the findings should not be generalized uncritically to conservatory students, professional pianists, younger learners, or students in non-sport universities. The sport-university context was treated as a bounded study setting rather than a directly measured explanatory variable. Because the study did not include direct measures of sport-university culture or a comparison group of conservatory students, it cannot determine whether sport-university culture itself explains the observed associations. Future comparative studies could examine whether the same regulatory configurations appear among conservatory students, general university music students, and professional performers.

Fifth, recruitment relied on a multi-site purposive online sample with snowball-assisted dissemination. This approach was appropriate for reaching eligible art students across multiple sport universities, but it may have introduced self-selection bias. Students who were more interested in piano performance, more engaged in art training, or more socially connected may have been more likely to participate. However, this remains a potential limitation of population representativeness rather than demonstrated evidence that the internal association pattern was invalid. Future studies should use probability-based sampling, institutional sampling frames, or stratified sampling procedures when possible.

Sixth, the fsQCA results depend on calibration choices and analytical thresholds. The study conducted robustness checks by adjusting frequency thresholds, PRI thresholds, consistency thresholds, and calibration anchors, and the core interpretation remained generally stable. Nevertheless, QCA methodologists emphasize that configurational results should be interpreted in relation to calibration decisions, case knowledge, and the theoretical meaning of set membership (Skaaning, 2011; Thomann and Maggetti, 2020). Future research could strengthen the configurational explanation by combining fsQCA with qualitative interviews or case-based performance narratives, especially to understand why some students fit one high-flow profile rather than another.

Finally, the present model focused on mindfulness, self-control, and cognitive emotion regulation, but other performance-related variables may further clarify piano performance flow. Music performance anxiety, self-efficacy, perfectionism, perceived challenge–skill balance, teacher support, and actual performance difficulty may interact with the regulatory variables examined here. Because no intervention was tested, the pedagogical implications should be treated as hypotheses for future research. Future studies should consider more fine-grained models that distinguish pre-performance preparation, in-performance regulation, and post-performance reflection. This would better capture the dynamic nature of flow as an optimal but fragile experience in piano performance.

## 5 Conclusion

This study examined piano performance flow experience among art students at Chinese sport universities from a psychological regulatory perspective by integrating SEM-based net-association analysis and fsQCA configurational analysis. The findings indicate that piano performance flow was closely associated with mindfulness, self-control, adaptive cognitive emotion regulation, and maladaptive cognitive emotion regulation, with maladaptive cognitive emotion regulation showing a particularly important role across the SEM, bootstrap, and fsQCA results. The SEM findings showed that mindfulness was directly associated with piano performance flow and indirectly associated with it through self-control and two forms of cognitive emotion regulation, while the direct association between self-control and flow was not statistically significant after the other regulatory variables were included. The fsQCA findings further showed that high flow was linked to two configurations—high mindfulness, high self-control, and low maladaptive cognitive emotion regulation; and high mindfulness, high adaptive cognitive emotion regulation, and low maladaptive cognitive emotion regulation—whereas non-high flow was linked to the combination of low mindfulness, low adaptive cognitive emotion regulation, and high maladaptive cognitive emotion regulation.

Together, these results suggest that piano performance flow among art students at sport universities is better understood not as being characterized by a single psychological resource alone, but as an optimal performance experience associated with specific patterns of attentional awareness, self-regulation, and cognitive emotion regulation. Given the cross-sectional, retrospective, and self-report design, these findings should be interpreted as associational and hypothesis-generating rather than as evidence of causal, real-time, or intervention effects.

## Data Availability

The original contributions presented in the study are included in the article/Supplementary material, further inquiries can be directed to the corresponding author.
